# A jackknife approach to estimate the prediction uncertainty from binary classifiers under right-censoring

**DOI:** 10.1177/09622802251393626

**Published:** 2025-11-14

**Authors:** Antje Jahn-Eimermacher, Lukas Klein, Gunter Grieser

**Affiliations:** 1 Department of Mathematics and Natural Sciences, University of Applied Sciences, Darmstadt, Germany; 2 European University of Technology, European Union, Darmstadt, Germany; 3 Faculty of Medicine, Martin-Luther-University Halle-Wittenberg, Halle (Saale), German; 4 Department of Computer Science, University of Applied Sciences, Darmstadt, Germany

**Keywords:** Prediction, survival analysis, uncertainty, machine learning, inverse-probability-of-censoring

## Abstract

Clinical prediction models are developed to estimate a patient’s risk for a specific outcome, and machine learning is frequently employed to improve prediction accuracy. When the outcome is some event that happens over time, binary classifiers can predict the risk at specific time points if right-censoring is addressed by inverse-probability-of-censoring-weighting . Assessing prediction uncertainty is crucial for interpreting individual risks, but there is limited knowledge on how to consider inverse-probability-of-censoring-weighting when estimating this uncertainty. We propose an adjustment of the infinitesimal jackknife estimator for the standard error of predictions that incorporates inverse-probability-of-censoring-weighting. By using a nonparametric approach, it is broadly applicable, especially to machine learning classifiers. For a simple tractable example, we show that the proposed adjustment reveals unbiased standard error estimates. For other situations, we evaluate performance through simulation studies under both parametric models with inverse-probability-of-censoring-weighting-customized log-likelihood and machine learning with inverse-probability-of-censoring-weighting-customized loss function. We illustrate the methods by predicting post-transplant survival probabilities, using national kidney transplant registry data. Our findings show that the proposed estimator is useful for quantifying prediction uncertainty of inverse-probability-of-censoring-weighting classifiers. Applications to simulated and real data show that prediction uncertainty increases when employing binary classifiers on dichotomized data compared to predictions from survival models.

## 1. Introduction

There is a significant interest in machine learning for predicting a patient’s long-term outcome after some medical intervention. An example motivating this paper is the patients’ and organ survival rate after organ transplantation.^1–3^ Event time data are subject to censoring when patients enter the study cohort at different times, resulting in varying lengths of follow-up and/or when patients prematurely leave the study cohort and become lost to follow-up. Numerous survival analysis methods address censored event time data and many of them model the hazard function.

In the field of machine learning, it is more common to predict the risk or survival probability at a specific time point, denoted as τ . Instead of relying on survival methodology, which involves modeling hazards, binary classifiers are often used for this purpose.^4–7^ These classifiers dichotomize the event time outcome into a binary variable indicating whether a subject has survived up to time τ or not. However, the dichotomized outcome remains unknown for subjects that are lost to follow-up both before time τ and before experiencing the event of interest. Reps et al.^8^ and Kvamme and Borgan^9^ have demonstrated that treating these censored observations as missing data in binary classifiers performs poorly in terms of discrimination and calibration. To address this issue, an alternative approach has been proposed:^10–12^ weighting the observations by their inverse-probability-of-censoring (IPC) and applying the classification algorithm to the IPC-weighted observations.

Predictions obtained from IPC-weighted binary classifiers have been demonstrated to be asymptotically unbiased employing concepts of maximum likelihoods^10,12^ or statistical functionals^11,13^ and some applications show promising results.^11,14^ However, existing research has primarily focused on bias, neglecting the investigation of prediction uncertainty under IPC-weighted classification. Prediction uncertainty arises from using a random sample from the target population. Binary classifiers derive their results from dichotomized sample data and thus do not use the full available information on event times. Consequently, IPC-weighted classification may introduce higher uncertainties in predicted survival probabilities compared to survival methodologies, such as (semi-)parametric survival regression, random survival forests^15^ or hazard-based neural networks.^16^

However, how to consider the IPC weights when estimating the prediction uncertainty of inverse-probability-of-censoring-weighting (IPCW) classifiers is—to the best of our knowledge—unknown. Available methods are rare and focus on selected parametric models.^12^ Targeting specific parametric models when estimating the uncertainty of predictions is common^17,18^ but limits its applicability in particular for machine learning. In this article, we will, therefore, first adjust the nonparametric infinitesimal jackknife estimator for the standard error of predictions to the IPC weights. Afterwards, this estimator will be employed to compare the prediction uncertainty of survival probabilities when derived from IPC-weighted classifiers compared to hazard-based algorithms.

Our investigation complements existing research on prediction accuracy of IPC-weighted binary classifiers with censored data by an investigation of prediction uncertainty. It will help to balance its potential disadvantage that results from using less information from the data against its potential benefits that can be summarized as follows: First, classification tasks are common in machine learning and artificial intelligence and thus novel algorithms often emerge first for classification (and regression) before adaptations to event time data are developed. For the same reason, widely used machine learning and deep learning libraries, such as scikit-learn and Keras, as well as automated machine learning (AutoML) solutions like H2O AutoML and SageMaker Autopilot/Canvas, integrate models and metrics for both classification and regression tasks, but not event time data. Second, the data structure itself can motivate a binary classifier. For example when events are recorded only annually (as in some registries or healthcare databases), estimating annual survival probabilities using binary classifiers can be reasonable. Third, binary classifiers can be appealing for their simplicity: when the interest lies in a single timepoint (e.g. annual survival probabilities) binary classifiers provide results that are easier to communicate than for example, hazard ratios, which may not always be the most intuitive measure of effect size.^12,19^

As an example the proposed methods are used to quantify the prediction uncertainty of machine learning classifiers when predicting the individual patient risks of death or graft failure after kidney transplantation. Kidney transplantation can be considered as the treatment of choice for patients with end-stage kidney disease, due to its potential for improved quality of life and longer life expectancy. However, the demand for donor kidneys far exceeds the organs available for donation, leading to important questions, for example, about the patients’ post-transplant prognosis.

The article is structured as follows: In Section 2, the infinitesimal jackknife estimator with an adjustment for IPCW is proposed, followed by an evaluation of its performance through simulation studies in Section 3. In Section 4, we apply the proposed estimator to assess prediction uncertainty when analyzing data from the German Organ Transplant Registry. Finally, Section 5 concludes with a discussion.

## 2. Methods

We first introduce some notations needed throughout this article: For i=1…n individuals, realizations of the random variables $\left(X_{i},T_{i},\delta_{i}\right)$ are observed that are considered as independent identically distributed copies of (X,T,δ) . Thereby, $T:=T^{*}\wedge C$ denotes the potentially right-censored event time with $T^{*}$ and C defining the time to event and the time to censoring, respectively. The symbol ∧ is used to define the minimum throughout the article. The random variable $\delta =I(T^{*}\leq C)$ , with I defining the indicator function, informs whether T defines an event or censoring time. The random baseline covariate vector X describes subject characteristics that might affect the survival probabilities over time. Thus, for a single observation i , $x_{i}$ defines the p-dimensional vector of covariate values, $t_{i}$ defines the time to event or censoring and $\delta_{i}$ defines the indicator whether an event has been observed at time $t_{i}$ . We assume that $T_{i}^{*}$ and $C_{i}$ are independent conditional on $X_{i}$ .

We are now interested in predicting the probability $p_{i}$ to survive a certain time point τ for subject i . This probability is defined as follows:

$$p_{i}:=P(T^{*}\geq \tau |X=x_{i})=P(Y=1|X=x_{i})$$

The random variable $Y:=I(T^{*}\geq \tau )$ represents the dichotomized outcome that a binary classifier could use as the dependent variable for predicting $p_{i}$ . When there is no censoring, $Y=I(T^{*}\geq \tau )$ is observable for all subjects and we can use any prediction model for classification tasks to estimate the prognosis $p_{i}$ . However, in the presence of censoring, for observations where $c_{i}<t_{i}^{*}\wedge \tau$ , the information about surviving τ and thus about Y is missing. Ad hoc approaches that build a classification model using only observations with available information on Y will yield biased predictions of $p_{i}$ .^8,9^ This bias arises because the distribution of $T^{*}$ conditional on $C\geq T^{*}\wedge \tau$ is obviously not the same as the distribution of $T^{*}$ , and the same holds for Y .

Weighting observations by their IPC has been proposed to prevent bias^10–12^ and is investigated in the present article. The IPC weights are defined as follows:

$${\tilde{w}}_{i}=\left\{ \begin{array}{ll} 0, & c_{i}<\tau \wedge t_{i}^{*}\\ \frac{1}{P(C>\tau |X=x_{i})}, & \tau <c_{i}\wedge t_{i}^{*}\\ \frac{1}{P(C>t_{i}|X=x_{i})}, & t_{i}^{*}<c_{i}\wedge \tau \end{array}\right.$$

Assigning the normalized weights $w_{i}:={\tilde{w}}_{i}/\sum_{k=1}^{n}{\tilde{w}}_{i}$ to the observations of the learning sample will assign weight zero to observations with unknown $Y_{i}$ . These discarded observations will then be represented by the observations to which greater weights were assigned. The use of IPC weights in classification algorithms can give unbiased predictions as shown in the following section.

### 2.1. Accuracy of predictions under IPC-weighted classification

First, we will confirm that any binary classifier that employs the IPC-weighted binary log loss function yields unbiased predictions of $p_{i}$ . This, for example, refers to generalized linear models with logit link function where maximizing the log-likelihood with IPC-weighted contributions is equivalent to minimizing the weighted binary log loss function. Another example is gradient boosting or neural networks with customized weighted binary log loss function. To demonstrate this, consider a binary classifier that seeks to find a prediction model π:X→Y that minimizes the expected IPC-weighted binary log loss function, $E[loss_{w}(\tau )]$ . Thereby, the expected value is considered as conditional on the covariates and refers to the distribution of new data that are independent of the training sample. For simplicity, we assume that P(C>u) is a known function in u , although in practice it needs to be estimated. The proof builds upon ideas from Kvamme and Borgan^9^ for calculating the expected value of IPCW-Brier scores. It can complement other theoretical justifications of IPC-weighted classification that rely on maximum likelihood theory^10^ or on classification considered as functionals in the data distribution.^11,13^

$$\begin{array}{rl} E[loss_{w}(\tau )] & =E\left[-\sum_{i=1}^{n}w_{i}(I(Y_{i}=1)\ln\;(p_{i})+I(Y_{i}=0)\ln\;(1-p_{i}))\right]\\ & =E[-\sum_{i=1}^{n}(\frac{1}{P(C>\tau |X=x_{i})}I(T_{i}^{*}\geq \tau ,C_{i}>\tau )\ln\;(p_{i}))\\ & \;-\sum_{i=1}^{n}(w_{i}I(T_{i}^{*}<\tau ,\delta_{i}=1)\ln\;(1-p_{i}))]\\ & =-\sum_{i=1}^{n}\frac{1}{P(C>\tau |X=x_{i})}P(T^{*}\geq \tau ,C>\tau |X=x_{i})\ln\;(p_{i})\\ & \;-\sum_{i=1}^{n}\int_{0}^{\tau }\frac{1}{P(C>u|X=x_{i})}f_{T^{*}|X=x_{i}}(u)P(C>u|X=x_{i})du\ln\;(1-p_{i})\\ & =-\sum_{i=1}^{n}(P(T^{*}\geq \tau |X=x_{i})\ln\;(p_{i})+P(T^{*}<\tau |X=x_{i})\ln\;(1-p_{i})) \end{array}$$

This is the average cross-entropy across all data samples and thus reaches its minimum at $p_{i}=P(T^{*}\geq \tau |X=x_{i})$ according to Gibb’s inequality. Consequently, the classifier that minimizes the weighted loss is expected to predict $p_{i}=P(T^{*}\geq \tau |X=x_{i})$ , resulting in unbiased predictions. After having confirmed that IPC-weighted classification can yield accurate predictions, we will now investigate the uncertainty of predictions. An available method on standard error estimation for predictions that are derived from IPC-weighted binary classifiers refers to generalized linear models,^12^ which limits its generalizability. Consequently, our next step is to investigate model-free approaches for estimating standard errors in the context of IPC-weighted classification.

### 2.2. Adjusting the jackknife standard error estimate to IPC weights

We start with the infinitesimal jackknife estimator for the standard error of predictions. By its nonparametric nature, jackknife estimators are not restricted to any particular classification algorithm. The infinitesimal jackknife standard error estimator is based on the concept of influence functions.^20^ To consider the IPCW within this concept, we introduce an adjustment as follows:

To simplify notation, we will omit the subject index when denoting the probability p to survive time τ in the following and we will use the indices to indicate jackknife replications instead. Let F be the (unknown) distribution where the observations Y are sampled from. The probability p to survive the time point τ is assumed to be some functional T of the distribution F , T(F) . The estimator of p is derived from the empirical distribution $\hat{F}$ , thus $\hat{p}=T(\hat{F})$ . In general, the influence function of T under distribution F is defined as follows:

(1) $$IF_{F}(y):=\lim_{\epsilon ↓0}\frac{T((1-\epsilon )F+\epsilon \delta_{y})-T(F)}{\epsilon }$$

with $\delta_{y}$ being the distribution with all its mass on y . The influence function can be used for a standard error estimate of $\hat{p}$ .^21^ With $IF_{F}(Y)$ being the influence function for some random observation Y sampled from F , the variance of $\hat{p}$ is then estimated by

(2) $${\hat{Var}}^{IJK}(\hat{p})=\frac{1}{n}\hat{Var}(IF_{F}(Y))=\frac{1}{n}\frac{1}{n-1}\sum_{i=1}^{n}(IF_{\hat{F}}(y_{i}){)}^{2}=\sum_{i=1}^{n}\frac{1}{n}\frac{\frac{1}{n}}{1-\frac{1}{n}}(IF_{\hat{F}}(y_{i}){)}^{2}$$

In the unweighted situation, the sample weights of the empirical distribution are 1/n for each observation $y_{i}$ . In the weighted situation, observations are differentially weighted by the IPC weights $w_{i}$ . We will, therefore, adjust the variance estimator (2) by replacing 1/n with $w_{i}$ . Furthermore, in the unweighted situation the assumption $IF_{\hat{F}}(y_{i})=(n-1)(\hat{p}-{\hat{p}}_{-i})$ is commonly used with ${\hat{p}}_{-i}$ being the estimate of p when omitting observation i from the sample. This results in the jackknife variance estimate $\frac{n-1}{n}\sum_{i=1}^{n}(\hat{p}-{\hat{p}}_{-i}{)}^{2}$ . However, this association might no longer be true in the weighted situation, in particular as the influence of an observation $Y_{i}$ might depend on its weight $w_{i}$ . We will illustrate this for an example in Section 2.3. We instead assume the following weighted association between influence components and jackknife replications ${\hat{p}}_{-i}$ for observations with $w_{i}\neq 0$ :

(3) $$IF_{\hat{F}}(y_{i})=\left(\frac{1}{w_{i}}-1\right)(\hat{p}-{\hat{p}}_{-i})$$

We will show for the analytically tractable example in Section 2.3 that this assumption holds true. Equation (3) has been shown also for more complex scenarios.^22^ Finally, these two adjustments result in the following adjusted infinitesimal jackknife standard error estimate of $\hat{p}$

(4) $$\begin{array}{rl} \hat{Var}(\hat{p})= & \sum_{i=1}^{n}w_{i}\frac{w_{i}}{1-w_{i}}(IF_{\hat{F}}(y_{i}){)}^{2}=\sum_{\begin{array}{c} i=1\\ w_{i}\neq 0 \end{array}}^{n}w_{i}\frac{w_{i}}{1-w_{i}}{\left(\frac{1}{w_{i}}-1\right)}^{2}(\hat{p}-{\hat{p}}_{-i}{)}^{2}\\ = & \sum_{i=1}^{n}(1-w_{i})(\hat{p}-{\hat{p}}_{-i}{)}^{2} \end{array}$$

Observations with $w_{i}=0$ are assigned weight zero in the first sum and, therefore, do not contribute to the sum. They are excluded in the second sum only to well define $1/w_{i}$ , that is no longer required in the third sum because $\hat{p}={\hat{p}}_{-i}$ for observations with weight zero.

#### 2.2.1. Relationship to delete-d jackknife estimators

The proposed estimator can be considered as a generalization of the delete-d jackknife variance estimate^23^ defined as

(5) $$\frac{n-d}{d}\frac{1}{\left(\begin{array}{l} n\\ d \end{array}\right)}\sum_{S_{d}\subseteq \{1,\ldots ,n\}}(\hat{p}-{\hat{p}}_{-S_{d}}{)}^{2}$$

with $S_{d}$ defining a subset of size d and ${\hat{p}}_{-S_{d}}$ defining the estimator of *p* that results when $S_{d}$ is left out from the training data. Thus, in contrast to ${\hat{p}}_{-i}$ , when estimating ${\hat{p}}_{-S_{d}}$ , not only 1 but d>1 observations are left out. In total, $\left(\begin{array}{l} n\\ d \end{array}\right)$ different left-out-subsets of size *d*, $S_{d}$ , exist and the resulting estimates ${\hat{p}}_{-S_{d}}$ are averaged with respect to their squared difference to $\hat{p}$ .

In the weighted situation, we no longer consider d but $nw_{i}$ as the amount of information that is left out when estimating $(\hat{p}{)}_{-i}$ . Consequently, the term (n−d)/d in (5) can be replaced by $\left(n-nw_{i}\right)/nw_{i}$ . Furthermore, we no longer average over $\left(\begin{array}{l} n\\ d \end{array}\right)$ delete-d estimators. Consequently, averaging over the $\left(\begin{array}{l} n\\ d \end{array}\right)$ squared differences can be replaced by the weighted mean of squared differences. Both replacements then result in the same estimator as proposed in (4) as follows:

$$\sum_{i=1}^{n}\frac{n-nw_{i}}{nw_{i}}w_{i}(\hat{p}-{\hat{p}}_{-i}{)}^{2}=\sum_{i=1}^{n}(1-w_{i})(\hat{p}-{\hat{p}}_{-i}{)}^{2}=\hat{Var}(\hat{p})$$

### 2.3. An analytically tractable example

We will now substantiate our adjustment using the weighted mean as a simple, analytically tractable example. The weighted mean of the dichotomized outcomes $y_{k}$ is the simplest classifier for estimating p , ignoring any covariate information during prediction. Consequently, it predicts the same probability $\hat{p}$ for each subject. This prediction will be $\hat{p}=\sum_{k=1}^{n}w_{k}Y_{k}$ . In other words, $\hat{p}=\int xd\hat{F}(x)=T(\hat{F})$ , where $\hat{F}$ is the empirical distribution of Y putting mass $w_{i}$ on each observed value $y_{i}$ . This is used as an estimate of $T(F)=\int xdF(x)=E(Y)=P(Y=1)=P(T^{*}\geq \tau )$ with F denoting the true distribution of Y . The influence components for an observation with $w_{i}\neq 0$ will be

$$\begin{array}{rl} IF_{\hat{F}}(y_{i}) & =\lim_{\epsilon ↓0}\frac{(1-\epsilon )\sum_{k=1}^{n}w_{k}y_{k}+\epsilon y_{i}-\sum_{k=1}^{n}w_{k}y_{k}}{\epsilon }\\ & =y_{i}-\sum_{k=1}^{n}w_{k}y_{k}\\ & =\frac{1}{w_{i}}\left(-\sum_{k=1}^{n}w_{k}y_{k}+w_{i}y_{i}+(1-w_{i})\sum_{k=1}^{n}w_{k}y_{k}\right)\\ & =\frac{1-w_{i}}{w_{i}}\left(\sum_{k=1}^{n}w_{k}y_{k}-\sum_{k\neq i}\frac{w_{k}}{1-w_{i}}y_{k}\right)\\ & =\left(\frac{1}{w_{i}}-1\right)(\hat{p}-{\hat{p}}_{-i}) \end{array}$$

with ${\hat{p}}_{-i}=\sum_{k\neq i}\frac{w_{k}}{1-w_{i}}y_{k}$ and thus are an example where (3) instead of $IF_{\hat{F}}(y_{i})=(n-1)(\hat{p}-{\hat{p}}_{-i})$ holds.

Now consider a simplified two-point discrete distribution for both time to event and time to censoring

$$P(T^{*}=t)=\left\{ \begin{array}{ll} 1-p & \text{if }t=0.75\\ p & \text{if }t=1.25 \end{array}\right.\;\;P(C=c)=\left\{ \begin{array}{ll} 1-q & \text{if }c=0.5\\ q & \text{if }c=2 \end{array}\right.$$

Consider we are interested in $p=P(T^{*}\geq 1)=P(T^{*}\geq \tau )$ with τ=1 and want to estimate p from an i.i.d. random sample of size n using the IPC-weighted mean. Let n−m be the number of observations that are censored at c=0.5 and thus receive a weight of 0. The other weights are estimated from the data and since $\hat{P}(C>t_{i})=\hat{P}(C>\tau )=(m/n)$ for i=1⋯n the normalized weight $w_{0}:=1/m$ is assigned to the remaining *m* observations. W.l.o.g., let the index set {i:i=1…m} refer to the observations that receive the non-zero weight $w_{0}$ . The weighted mean is then given by $\hat{p}=\frac{1}{m}\sum_{i=1}^{m}Y_{i}$ with well-known variance $\frac{1}{m}p(1-p)$ . The adjusted jackknife estimator of prediction uncertainty we have proposed in (4) then is

$$\begin{array}{rl} \hat{Var}(\hat{p}) & =\sum_{i=1}^{n}(1-w_{i})\cdot (\hat{p}-{\hat{p}}_{-i}{)}^{2}\\ & =\sum_{i=1}^{n}(1-w_{i}){\left(\frac{w_{i}}{1-w_{i}}(y_{i}-\hat{p})\right)}^{2}\\ & =\frac{1}{m}\frac{1}{m-1}\sum_{i=1}^{m}(y_{i}-\hat{p}{)}^{2} \end{array}$$

Thus, in this simplified scenario, the proposed estimator simplifies to the unbiased sample variance estimator of the mean of m i.i.d. random variables.

#### 2.3.1. Comparison to the unadjusted jackknife and Greenwood’s standard error estimate

The unadjusted jackknife standard error estimate $\sum_{i=1}^{n}\frac{n-1}{n}(\hat{p}-{\hat{p}}_{-i}{)}^{2}$ is a scaled version of the unbiased sample variance with scaling factor $\frac{m}{m-1}\frac{n-1}{n}$ and thus approximates the adjusted estimator with increasing n and m . We can also compare the adjusted jackknife estimator with another well-known variance estimate in this simplified scenario. The IPC-weighted mean corresponds to the Kaplan-Meier estimate at τ , ${\hat{S}}_{KM}$ , which can be written as a weighted sum.^24^ Therefore, Greenwood’s variance estimate, ${\hat{Var}}_{GW}(\hat{S}(\tau ))$ , would be an alternative estimator that turns out to be a scaled version of the unbiased adjusted jackknife estimate:

$$\begin{array}{rl} {\hat{Var}}_{GW}(\hat{S}(\tau )) & =\frac{d(m-d)}{m^{3}}\\ & =\frac{1}{m^{2}}\left(d{\left(1-\frac{d}{m}\right)}^{2}+(m-d){\left(\frac{d}{m}\right)}^{2}\right)\\ & =\frac{1}{m^{2}}\sum_{i=1}^{m}(y_{i}-\hat{p}{)}^{2}\\ & =\frac{m-1}{m}\hat{Var}(\hat{p}) \end{array}$$

with $d=m-\sum_{i=1}^{m}y_{i}$ being the number of observed events before τ .

### 2.4. Using standard error estimates to calculate confidence intervals

Under certain distributional assumptions on the prediction estimate of interest, $\hat{p}$ , standard error estimates can be used to construct an 100(1−α) % confidence interval for the survival probability p . If $\hat{p}$ can be assumed to be (asymptotically) normally distributed, a Wald-type confidence interval can be derived as $\hat{p}\pm z_{\alpha /2}SE(\hat{p})$ with $SE(\hat{p})=\hat{\text{Var}}(\hat{p}{)}^{1/2}$ . However, this approach can yield confidence intervals with boundaries outside the range [0,1] , which is not reasonable for probabilities. To address this issue, an alternative approach transforms the boundaries of a confidence interval calculated on the logit scale,^25^ which ensures that the resulting interval lies within [0, 1]. This method still relies on the assumption of asymptotic normality of $\hat{p}$ and uses the delta method to derive the confidence interval on the logit scale. The confidence interval is then given by the following equation:

(6) $$\left[\frac{\exp\;(LL_{logit})}{1+\exp\;(LL_{logit})};\frac{\exp\;(UL_{logit})}{1+\exp\;(UL_{logit})}\right]$$

where $LL_{logit}$ and $UL_{logit}$ are the lower and upper bounds of the 100(1−α) % confidence interval for the logit of $\hat{p}$ , respectively, which can be found by applying the delta method on the logit transformation of $\hat{p}$ :

$$LL_{logit}/UL_{logit}=\ln\;\left(\frac{\hat{p}}{1-\hat{p}}\right)\pm z_{\alpha /2}\frac{SE(\hat{p})}{\hat{p}(1-\hat{p})}$$

To inspect the asymptotic normality assumption of $\hat{p}$ , a specific analysis model and data-generating process would have to be defined, which was not required for the nonparametric jackknife approach for estimating the standard error. Investigating the asymptotic behavior of $\hat{p}$ is beyond the scope of the present article and has been done specifically for the IPC-weighted maximum likelihood solution of a generalized log linear model before.^12^ Instead, we will assess the coverage probability of the confidence interval given in (6) in a simulation study both with a parametric log linear model and a nonparametric gradient boosting approach.

## 3. Simulation study

In situations where less simple classifiers are applied, the adjusted jackknife variance estimator may no longer be analytically tractable and we will explore its properties through a simulation study. Our findings will be evaluated in a simulation study following the ADEMP structure.^26^ Applied analysis methods are available as an R package on GitHub (see the *Data availability* section).

### 3.1. Simulation design

#### Objective:

We aim to investigate finite-sample properties of the predictions derived from IPC-weighted classification algorithms together with finite-sample properties of the proposed adjusted jackknife standard error estimator and the confidence intervals. In particular, we compare the predictions $\hat{p}$ , their estimated standard error $(\hat{Var}(\hat{p}){)}^{1/2}$ and the coverage probability of the derived 100(1−α) % confidence interval for p with the true probability p to survive time τ=5 , the empirical standard deviation of predictions $\hat{p}$ and the nominal confidence level 100(1−α) %, respectively. In the first step, we use a simple parametric generalized linear classification model (GLM), because for the GLM a model-based standard error estimator exists as a benchmark. In the second step, a gradient boosting classifier will be applied with a customized IPC-weighted binary log-loss function. Here, no benchmark exists for the standard error of predictions and the adjusted jackknife estimator will be compared to the empirical standard deviation of predictions across the simulation runs. For comparison, we will also apply a survival model that uses information on (T,C) instead of the dichotomized IPC-weighted outcome.

#### Methods:

We evaluate event time scenarios with sample size n∈{200,500,1000,5000} . For each sample size, we examine censoring distributions where 25% and 50% of the observations are expected to have a weight of 0, respectively. For each subject i , the values of two binary covariates, $x_{i1}\in \{-1,1\}$ and $x_{i2}\in \{0,1\}$ , are drawn, respectively, from independent B(1,0.5) -distributions. Time to event $t_{i}^{*}$ is generated from a log-logistic distribution with shape parameter a=1 and scale parameter $\exp\;(b_{0}+b_{1}x_{i1}+b_{2}x_{i2})$ with the true values of the regression coefficients being set to $b_{0}=1,b_{1}=-0.5,b_{2}=0.5$ . Time to censoring $c_{i}$ is generated from an exponential distribution with parameter $\lambda_{cens}\in \{0.017,0.10,0.27\}$ to ensure an average proportion of 5% , 25% , and 50% of observations receiving weight zero, respectively. Applying a log-logistic distribution ensures that the dichotomized outcome $Y=I(T^{*}\geq \tau )$ of the n independent realizations can be considered as a sample from a generalized linear model with logit link function. At the same time, $T^{*}$ follows a parametric survival model. Consequently, any differences in the prediction performance of both modeling strategies cannot be attributed to one model violating its modeling assumptions.

For the simulation, we use the rllogis function of the R-package flexsurv (version 2.2.2) to generate $n_{sim}=5000$ replications for each combination of n and $\lambda_{cens}$ . Each of these datasets is evaluated by the following three analysis methods:

(a) A parametric log-logistic survival model fitted using the survreg function from the R package survival (version 3.5.5). Predicted probabilities are obtained as follows:
$${\hat{p}}_{x}=\hat{P}(T^{*}>\tau |X=x)=(1+\exp\;(-(-\ln\;(\tau )+{\hat{b}}^{{}^{\prime }}x)){)}^{-1}$$
  where $\hat{b}$ is the maximum likelihood estimate of the model parameters. To simplify notation, we define the covariate vector x to include a leading 1, that is, $x=(1,x_{1},x_{2}{)}^{{}^{\prime }}$ .
(b) An IPC-weighted generalized linear model with logit link function fitted using the logitIPCW function of the R-package mets (version 1.3.3). Predicted probabilities are obtained as follows:
$${\hat{p}}_{x}=\hat{P}(Y=1|X=x)=(1+\exp\;(-(-\ln\;(\tau )+{\hat{b}}^{{}^{\prime }}x)){)}^{-1}$$
  where $\hat{b}$ minimizes the IPC-weighted loss (which is equivalent to maximizing the IPC-weighted log-likelihood):
$$\sum_{i=1}^{n}w_{i}\left(I(Y_{i}=1)\ln\;\left(\frac{\exp\;(b^{{}^{\prime }}x_{i})}{1+\exp\;(b^{{}^{\prime }}x_{i})}\right)+I(Y_{i}=0)\ln\;\left(\frac{1}{1+\exp\;(b^{{}^{\prime }}x_{i})}\right)\right)$$
  In particular, ${\hat{p}}_{x}$ can be considered as a functional $T(\hat{F})$ of the empirical distribution putting mass $w_{i}$ on each observed value $y_{i}$ , as this distribution serves as the basis for the loss function.
(c) An IPC-weighted gradient boosting approach fitted using the xgb.train function from the R-package xgboost (version 1.7.7). With this approach predictions cannot be expressed in closed form due to the nonparametric nature of the algorithm. However, as in (b), predictions are obtained by minimizing the IPC-weighted loss
$$\sum_{i=1}^{n}w_{i}(I(Y_{i}=1)\ln\;(p_{i})+I(Y_{i}=0)\ln\;(1-p_{i}))$$
  without an explicit parametric form of $p_{i}=P(Y=1|X=x_{i})$ . Nonetheless, ${\hat{p}}_{x}$ can still be considered as a functional $T(\hat{F})$ of the empirical distribution putting mass $w_{i}$ on each observed value $y_{i}$ , as this distribution still serves as the basis of the loss function.

For estimating the IPC weights that are passed to the classifiers in (b) and (c) we use a model-free Kaplan-Meier estimator. For each simulation run l , we extract the prediction estimates ${\hat{p}}_{kj}^{\left(l\right)}$ from each model fit. These estimates are for a subject with $x_{1}=k$ and $x_{2}=j$ , and they are derived as a function of the regression coefficient estimates in (a) and (b) and as the predicted outcome in (c). Model-based standard error estimates of predictions $(\text{mod}\text{-}\text{SE}({\hat{p}}_{kj}){)}^{\left(l\right)}$ can be derived in (a) and (b) from the standard error of regression coefficient estimates using the delta method. In addition, for all models the proposed adjusted jackknife standard error estimates $(\text{wJF}\text{-}\text{SE}({\hat{p}}_{kj}){)}^{\left(l\right)}$ are derived. Each standard error estimate is also used to compute a 100(1−α) % confidence interval following the approach given in (6). Furthermore, we estimate each model’s IPC-weighted Brier score $\text{wBS}=1/n\sum_{i=1}^{n}w_{i}({\hat{p}}_{i}-y_{i}{)}^{2}$ . Based on these data, we calculate the following measures of evaluation for each model in each scenario:

-
${\bar{p}}_{kj}$ : Empirical mean of the predictions ${\hat{p}}_{kj}^{\left(l\right)},l=1,\ldots ,n_{sim}$ .
-
${\text{SE}}_{kj}$ : Empirical standard deviation of ${\hat{p}}_{kj}^{\left(l\right)},l=1,\ldots ,n_{sim}$ .
-
$\bar{\text{BS}}$ : Empirical mean of the Brier scores ${\text{BS}}^{\left(l\right)},l=1,\ldots ,n_{sim}$ .
-
$\text{wJK}\text{-}{\text{SEE}}_{kj}$ : Empirical mean of the proposed model-free standard error estimates $(\text{wJK}\text{-}\text{SE}({\hat{p}}_{kj}){)}^{\left(l\right)},l=1,\ldots ,n_{sim}$ .
-
$\text{mod}\text{-}{\text{SEE}}_{kj}$ : Empirical mean of the model-based standard error estimates $(\text{mod}\text{-}\text{SE}({\hat{p}}_{kj}){)}^{\left(l\right)},l=1,\ldots ,n_{sim}$ (available for (a) and (b) only).
-
$\text{wJK}\text{-}{\text{CP}}_{kj}$ : Empirical coverage probability of the CI, calculated by (6) and using the proposed model-free standard error estimates. Empirical coverage probability is defined as the proportion of simulations in which the CI contains the true $p_{kj}$ .
-
$\text{mod}\text{-}{\text{CP}}_{kj}$ : Empirical coverage probability of the CI, calculated by (6) and using the model-based standard error estimates (if available). Empirical coverage probability is defined as the proportion of simulations in which the CI contains the true $p_{kj}$ .

### 3.2. Simulation results

As an example, we show the results of the prediction estimates ${\hat{p}}_{11}$ and ${\hat{p}}_{10}$ for a subject with $\left(x_{1},x_{2})=(1,1\right)$ and $\left(x_{1},x_{2})=(1,0\right)$ , respectively. The true probability of surviving time τ=5 is $p_{11}=0.352$ and $p_{10}=0.248$ , respectively. The mean predictions over the simulation runs are provided in Tables 1 and 2 for the different sample sizes and censoring rates investigated. The parametric survival model, which does not require IPCW, provides unbiased predictions as expected. Unbiased predictions are also achieved by the generalized linear model and the gradient boosting, that both employ the IPC-weighted log-loss function. This finding confirms our analytical results derived in Section 2.1. However, both appear to need a substantial number of observations with non-zero weights to produce unbiased predictions. While the IPC-weighted GLM stabilizes with n=500 observations, up to 50% of which may have zero weight, weighted gradient boosting stabilizes not before n=1000 (for $p_{11}$ ) or even n>1000 (for $p_{10}$ ), in particular when up to half of the observations have zero weight. Severe bias is observed for the XGBoost even with sample sizes up to *n*=1000 for $p_{10}$ and only with large sample sizes of n=5000 do these predictions become approximately unbiased. One reason might be that the machine learning method often performs better with higher number of covariates and more hyperparameter tuning might lead to better predictions. However, this result also underscores that the results presented in Section 2.1 refer to expected bias only, and that finite-sample behaviors may still differ—particularly when using data-demanding machine learning methods.

**Table 1.** Mean predicted probabilities to survive time point τ=5 for an individual with $x_{1}=1$ and $x_{2}=1$ ( ${\bar{p}}_{11}$ ) in simulated data with sample size *n*.

	% of obs	Parametric	IPCW-generalized	IPCW-gradient
*n*	with *w* = 0	survival model	linear model	boosting
200	5	0.353	0.352	0.361
200	25	0.353	0.351	0.366
200	50	0.352	0.346	0.385
500	5	0.351	0.351	0.352
500	25	0.351	0.351	0.350
500	50	0.352	0.349	0.359
1000	5	0.352	0.352	0.351
1000	25	0.352	0.352	0.350
1000	50	0.352	0.351	0.351
5000	5	0.352	0.352	0.352
5000	25	0.352	0.352	0.352
5000	50	0.352	0.352	0.351

The true survival probabaility is $p_{11}=0.352$ . The expected proportion of observations with assigned IPC weight of 0 is 5%, 25%, and 50% depending on the censoring distribution. IPC: inverse-probability-of-censoring; IPCW: inverse-probability-of-censoring-weighting.

**Table 2.** Mean predicted probabilities to survive time point τ=5 for an individual with $x_{1}=1$ and $x_{2}=0$ ( ${\bar{p}}_{10}$ ) in simulated data with sample size *n*.

	% of obs	Parametric	IPCW-generalized	IPCW-gradient
*n*	with *w* = 0	survival model	linear model	boosting
200	5	0.248	0.248	0.291
200	25	0.248	0.248	0.305
200	50	0.247	0.244	0.341
500	5	0.248	0.249	0.265
500	25	0.248	0.248	0.269
500	50	0.248	0.246	0.292
1000	5	0.248	0.248	0.256
1000	25	0.248	0.248	0.257
1000	50	0.248	0.248	0.269
5000	5	0.248	0.248	0.250
5000	25	0.248	0.248	0.250
5000	50	0.248	0.248	0.252

The true survival probabaility is $p_{10}=0.248$ . The expected proportion of observations with assigned IPC weight of 0 is 5%, 25%, and 50% depending on the censoring distribution. IPC: inverse-probability-of-censoring; IPCW: inverse-probability-of-censoring-weighting.

Tables 3 and 4 present the simulation results for the standard errors of ${\hat{p}}_{11}$ and ${\hat{p}}_{10}$ , respectively, estimated using the proposed IPCW-adjusted jackknife estimator (Section 2.2). For comparison, model-based standard error estimates are also shown, which are, however, not available for general machine learning approaches like XGBoost. The empirical standard errors over the simulation runs (SE) are expected to closely resemble the true standard error of predictions when derived from the different analysis methods and for different sample sizes and censoring rates. The model-based standard errors (mod-SEE) of the survival and IPC-weighted GLM are asymptotically unbiased and, consequently, closely match the empirical standard errors for larger sample sizes and lower censoring rates. The IPC-adjusted jackknife estimates for the IPCW-GLM model exhibit a bias of up to 6.2% relative to the empirical standard error estimate in certain scenarios ( n=200 , with 50% of observations having zero weight). However, in all cases where only 25% of observations have zero weight, the relative bias remains below 4%. For IPCW-weighted XGBoost, in scenarios where prediction bias arises due to insufficient sample sizes, the IPCW-based standard error estimates also exhibit bias, which can be substantial (up to 13.9% for $p_{11}$ and up to −34% for $p_{10}$ . Since the Jackknife variance estimator is based on predictions from leave-one-out subsamples, it is not surprising that biased predictions can lead to biased Jackknife standard errors. For sufficiently large sample sizes, when the bias of predictions diminishes, also the bias of the weighted Jackknife standard error estimates decreases to < 5.6% . Irrespective of the applied method, all standard error estimates increase with decreasing sample size and increasing censoring rates, because both lead to a reduced number of observed events.

**Table 3.** Standard error of the predicted probability to survive time point τ=5 for an individual with $x_{1}=1$ and $x_{2}=1$ ( $SE({\hat{p}}_{11})$ ) in simulated data with sample size n .

		Parametric		IPCW-generalized			IPCW-gradient	
		survival model		linear model			boosting	
	% of obs							
*n*	with *w*=0	SE	mod-SEE	SE	mod-SEE	wJK-SEE	SE	wJK-SEE
200	5	0.0508	0.0498	0.0618	0.0601	0.0612	0.0581	0.0662
200	25	0.0549	0.0539	0.0716	0.0696	0.0724	0.0715	0.0687
200	50	0.0622	0.0614	0.0997	0.0961	0.1058	0.0944	0.1011
500	5	0.0319	0.0316	0.0384	0.0381	0.0385	0.0371	0.0375
500	25	0.0348	0.0341	0.0446	0.0442	0.0454	0.0438	0.0442
500	50	0.0393	0.0389	0.0623	0.0615	0.0657	0.0613	0.0615
1000	5	0.0223	0.0224	0.0273	0.0270	0.0272	0.0271	0.0268
1000	25	0.0241	0.0242	0.0316	0.0313	0.0321	0.0314	0.0317
1000	50	0.0278	0.0275	0.0444	0.0437	0.0462	0.0431	0.0447
5000	5	0.0100	0.0100	0.0122	0.0121	0.0121	0.0123	0.0121
5000	25	0.0108	0.0108	0.0142	0.0140	0.0143	0.0142	0.0143
5000	50	0.0123	0.0123	0.0194	0.0196	0.0206	0.0194	0.0205

The expected proportion of observations with assigned IPC weight of 0 is 5%, 25%, and 50% depending on the censoring distribution. The empirical standard error over the simulation runs (SE), the mean standard error derived from standard error estimates of regression coefficients using the delta method (mod-SEE) and the mean adjusted jackknife standard error estimates (wJK-SEE) are given for the different prediction models. For gradient boosting no model-based standard error estimate exists, the survival model does not require IPCW and thus no adjusted jackknife estimator exists. IPC: inverse-probability-of-censoring; IPCW: inverse-probability of censoring weighting.

**Table 4.** Standard error of the predicted probability to survive time point τ=5 for an individual with $x_{1}=1$ and $x_{2}=0$ ( $SE({\hat{p}}_{10})$ ) in simulated data with sample size n .

		Parametric		IPCW-generalized			IPCW-gradient	
		survival model		linear model			boosting	
	% of obs							
*n*	with *w*=0	SE	mod-SEE	SE	mod-SEE	wJK-SEE	SE	wJK-SEE
200	5	0.0421	0.0421	0.0531	0.0524	0.0532	0.0709	0.0651
200	25	0.0458	0.0453	0.0623	0.0609	0.0630	0.0890	0.0593
200	50	0.0516	0.0512	0.0875	0.0843	0.0915	0.1160	0.1078
500	5	0.0264	0.0267	0.0329	0.0333	0.0336	0.0391	0.0314
500	25	0.0283	0.0287	0.0384	0.0388	0.0398	0.0477	0.0355
500	50	0.0328	0.0323	0.0550	0.0543	0.0575	0.0772	0.0507
1000	5	0.0190	0.0189	0.0238	0.0235	0.0237	0.0264	0.0224
1000	25	0.0204	0.0203	0.0279	0.0275	0.0281	0.0310	0.0263
1000	50	0.0231	0.0228	0.0392	0.0387	0.0407	0.0488	0.0357
5000	5	0.0084	0.0084	0.0105	0.0105	0.0106	0.0106	0.0104
5000	25	0.0091	0.0091	0.0122	0.0123	0.0125	0.0124	0.0123
5000	50	0.0101	0.0102	0.0173	0.0174	0.0182	0.0180	0.0177

The expected proportion of observations with assigned IPC weight of 0 is 5%, 25%, and 50% depending on the censoring distribution. The empirical standard error over the simulation runs (SE), the mean standard error derived from standard error estimates of regression coefficients using the delta method (mod-SEE) and the mean adjusted jackknife standard error estimates (wJK-SEE) are given for the different prediction models. For gradient boosting no model-based standard error estimate exists, the survival model does not require IPCW and thus no adjusted jackknife estimator exists. IPC: inverse-probability-of-censoring; IPCW: inverse-probability-of-censoring-weighting.

In general, the IPCW-adjusted jackknife estimates tend to slightly overestimate the standard errors. They can therefore be considered conservative, for example, with respect to coverage probabilities when applied to calculate confidence intervals of predictions. This is consistent with the results on coverage probabilities shown in Tables 5 and 6. Except for cases where sample size limitations introduce bias that adversely affects coverage (GLM with n=200 or with n=500 and 50% observations with zero weight and gradient boosting with n<1000 ( $p_{11}$ ) and n≤1000 ( $p_{10}$ ), the coverage probabilities using the IPCW-adjusted jackknife standard error come close to the nominal level of 95%. In general, they slightly exceed the nominal level, whereas those using the model-based standard error sometimes fall slightly below it. Overall, the IPCW-adjusted jackknife standard error seems to provide a useful method for constructing confidence intervals, particularly in situations where model-based solutions are not available. However, coverage probabilities of CI depend not only on reliable standard error estimates, but also on unbiased predictions. The latter explains the very poor coverage probabilities in certain scenarios with gradient boosting and n≤1000 , as shown in Table 6.

**Table 5.** Coverage probabilities of 95% confidence intervals for the probability to survive time point τ=5 for an individual with $x_{1}=1$ and $x_{2}=1$ in simulated data with sample size n .

		Parametric	IPCW-generalized		IPCW-gradient
	% of obs	survival model	linear model		boosting
*n*	with *w* = 0	mod-CP	mod-CP	wJK-CP	wJK-CP
200	5	0.950	0.945	0.949	0.918
200	25	0.951	0.949	0.957	0.888
200	50	0.952	0.948	0.967	0.812
500	5	0.947	0.948	0.950	0.948
500	25	0.945	0.950	0.955	0.950
500	50	0.948	0.952	0.964	0.925
1000	5	0.948	0.952	0.953	0.951
1000	25	0.948	0.950	0.955	0.952
1000	50	0.943	0.948	0.960	0.953
5000	5	0.955	0.945	0.946	0.945
5000	25	0.950	0.946	0.950	0.950
5000	50	0.948	0.954	0.963	0.962

The expected proportion of observations with assigned IPC weight of 0 is 5%, 25%, and 50% depending on the censoring distribution. The confidence intervals are derived from confidence intervals on the logit scale, that use the adjusted jackknife standard error (wJK-CP). For comparison, the coverage probabilities when using model-based standard error estimates from the parametric survival and generalized linear model are also given (mod-CP). IPC: inverse-probability-of-censoring; IPCW: inverse-probability-of-censoring-weighting.

**Table 6.** Coverage probabilities of 95% confidence intervals for the probability to survive time point τ=5 for an individual with $x_{1}=1$ and $x_{2}=0$ in simulated data with sample size n .

		Parametric	IPCW-generalized		IPCW-gradient
	% of obs	survival model	linear model		boosting
*n*	with *w* = 0	mod-CP	mod-CP	wJK-CP	wJK-CP
200	5	0.954	0.951	0.954	0.728
200	25	0.952	0.948	0.955	0.627
200	50	0.954	0.941	0.957	0.542
500	5	0.953	0.953	0.955	0.824
500	25	0.951	0.952	0.957	0.812
500	50	0.944	0.949	0.962	0.674
1000	5	0.948	0.947	0.949	0.878
1000	25	0.948	0.950	0.956	0.886
1000	50	0.950	0.948	0.959	0.804
5000	5	0.956	0.954	0.955	0.942
5000	25	0.952	0.954	0.957	0.945
5000	50	0.951	0.953	0.964	0.935

The expected proportion of observations with assigned IPC weight of 0 is 5%, 25%, and 50% depending on the censoring distribution. The confidence intervals are derived from confidence intervals on the logit scale, that use the adjusted jackknife standard error (wJK-CP). For comparison, the coverage probabilities when using model-based standard error estimates from the parametric survival and generalized linear model are also given (mod-CP). IPC: inverse-probability-of-censoring; IPCW: inverse-probability-of-censoring-weighting

In general, the construction of confidence intervals from the logit scale, as given in (6), can be recommended over Wald-type intervals, as they yield coverage probabilities closer to the nominal level of 95% (results for the Wald-type intervals not shown).

When only a small proportion—5%—of all subjects receive weight of zero, weighted approaches may suffer from increased uncertainty in estimating the censoring distribution, which determines the weights. Additionally, smaller weights can lead to numerically instability in the weighted estimators, as known from other IPCW-based approaches.^27^ However, our simulation results do not indicate any adverse effects resulting from small censoring proportions. One possible explanation is that a lower censoring rate implies fewer subjects with weight zero, that are represented by up-weighting remaining observations. This benefit might outweigh the potential drawbacks of smaller weights.

When comparing the prediction uncertainty between the different applied analysis models, the survival model exhibits the lowest degree of uncertainty when measured as the standard error of prediction. Survival models utilize the full information from the data, whereas classification models dichotomize the information, which could explain this result. However, it must be considered that the applied survival model perfectly fits the data generation process, whereas in real data applications, model misspecifications might also affect the prediction uncertainty. When comparing the IPC-weighted classifiers, the generalized linear model exhibits slightly higher prediction uncertainties than the gradient boosting. However, this is accompanied by a higher prediction accuracy, as described before.

Interestingly, the differences in prediction uncertainties seem to be too small to be reflected in the Brier scores presented in Table 7. Here, all methods show comparable results with respect to the Brier scores averaged over the simulation runs.

**Table 7.** Mean Brier score of the different prediction models for predicting the probability to survive time point τ=5 in simulated data with sample size *n*.

	% of obs	Parametric	IPCW-generalized	IPCW-gradient
*n*	with *w* = 0	survival model	linear model	boosting
200	5	0.2244	0.2227	0.2244
200	25	0.2242	0.2215	0.2239
200	50	0.2237	0.2170	0.2234
500	5	0.2255	0.2248	0.2251
500	25	0.2254	0.2243	0.2247
500	50	0.2248	0.2223	0.2239
1000	5	0.2257	0.2254	0.2254
1000	25	0.2257	0.2251	0.2252
1000	50	0.2256	0.2243	0.2247
5000	5	0.2261	0.2260	0.2260
5000	25	0.2261	0.2259	0.2260
5000	50	0.2261	0.2258	0.2258

The expected proportion of observations with assigned IPC weight of 0 is 5%, 25%, and 50% depending on the censoring distribution. IPC: inverse-probability-of-censoring; IPCW: inverse-probability-of-censoring-weighting.

**Figure 1.**
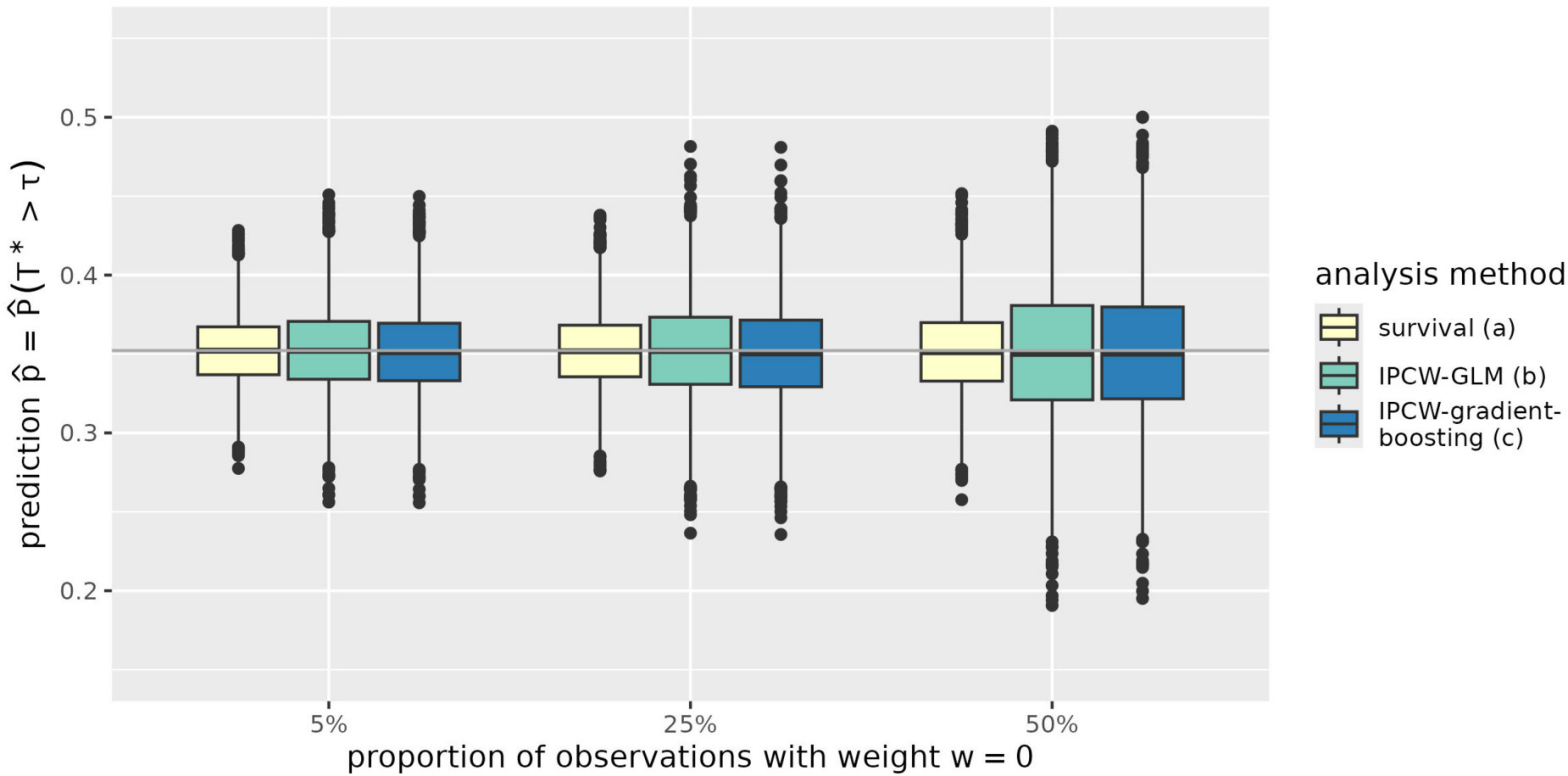
Predictions of the probability to survive time point τ=5 for an individual with $x_{1}=1$ and $x_{2}=1$ ( ${\hat{p}}_{11}$ ) in simulated data with sample size n=1000 . The expected proportion of observations with assigned IPC weight of 0 is 5%, 25%, and 50% depending on the censoring distribution. Predictions are derived from a parametric log-logistic survival model (survival), an IPCW-GLM and an IPC-weighted gradient boosting model (IPCW-gradient-boosting) described as (a) to (c) in Section 3.1. IPC: inverse-probability-of-censoring; IPCW-GLM: inverse-probability-of-censoring-weighted generalized linear model; IPCW: inverse-probability-of-censoring-weighting.

**Figure 2.**
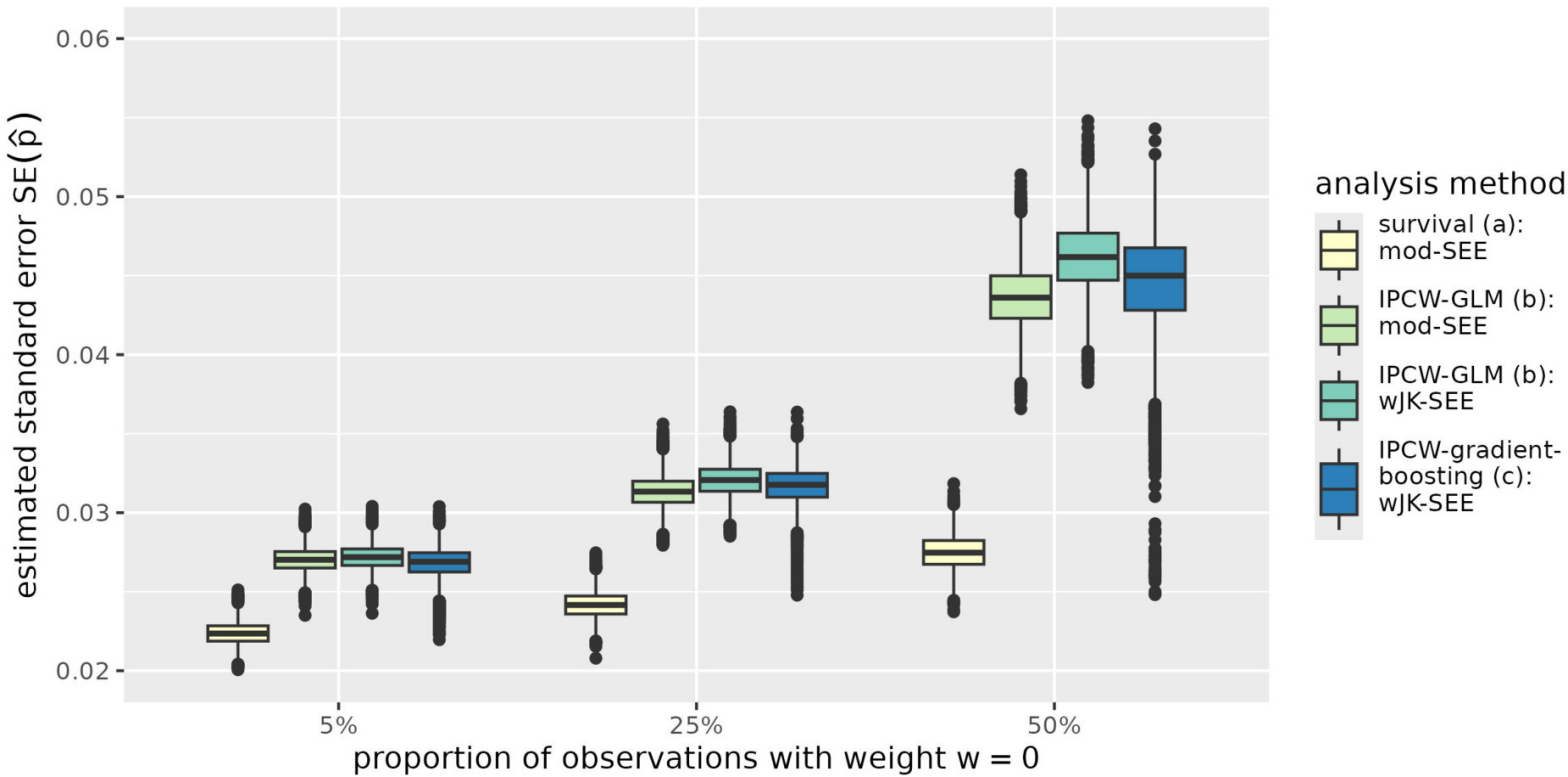
Standard error of the predicted probability to survive time point τ=5 for an individual with $x_{1}=1$ and $x_{2}=1$ ( ${\hat{p}}_{11}$ ) in simulated data with sample size n=1000 . The expected proportion of observations with assigned IPC weight of 0 is 5%, 25%, and 50% depending on the censoring distribution. Standard errors of predictions are derived from the standard errors of regression coefficients using the delta method in the parametric log-logistic survival model and the IPC-weighted-GLM (survival:mod-SEE, IPCW-GLM:mod-SEE) and by the adjusted jackknife estimator in the IPC-weighted-GLM and the IPC-weighted-gradient boosting model (IPCW-GLM:wJK-SEE, IPCW-gradient-bosting:wJK-SEE). The analysis methods (a) to (c) are described in Section 3.1. IPC: inverse-probability-of-censoring; IPCW-GLM: inverse-probability-of-censoring-weighted generalized linear model; IPCW: inverse-probability-of-censoring-weighting.

**Figure 3.**
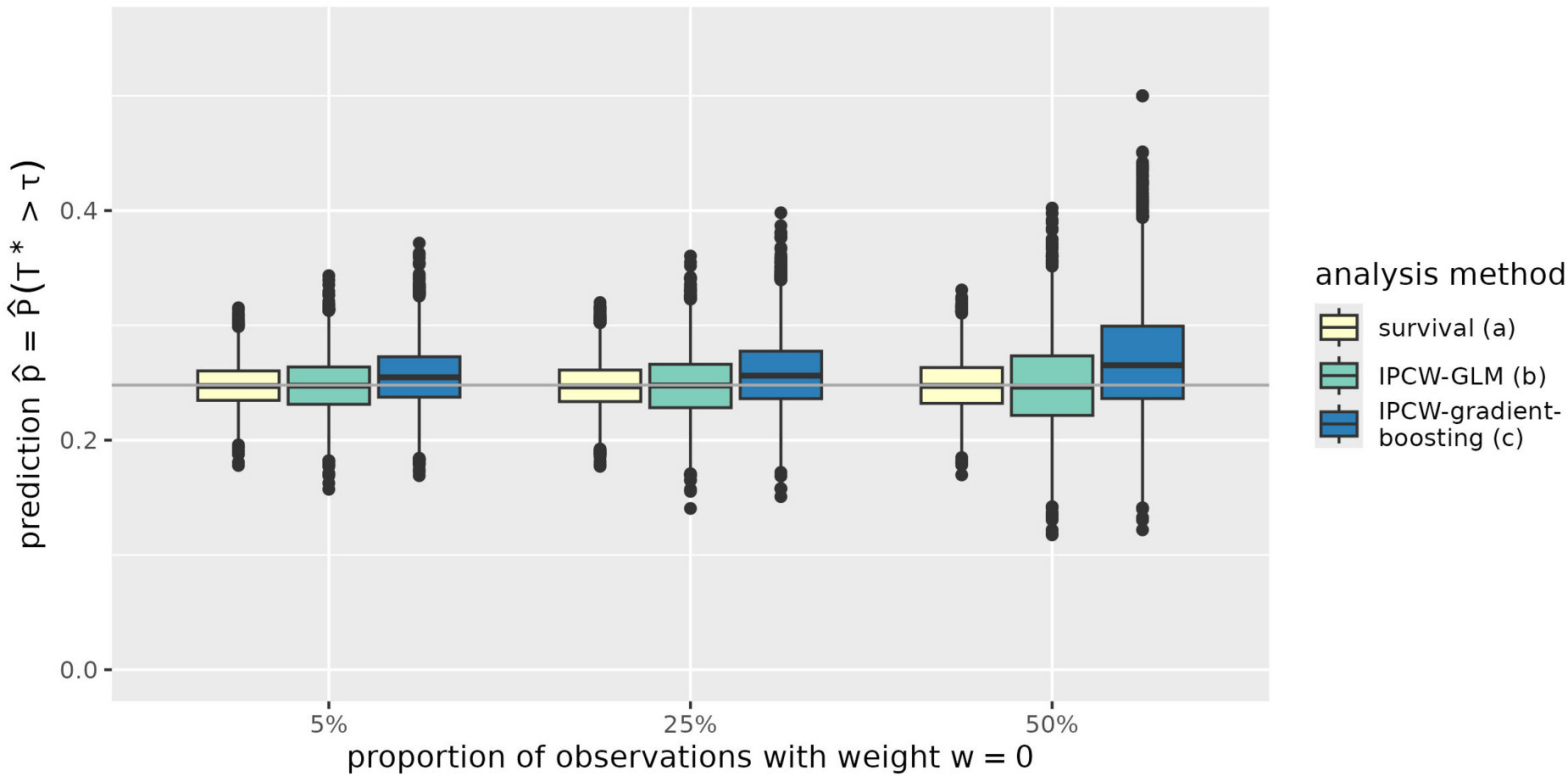
Predictions of the probability to survive time point τ=5 for an individual with $x_{1}=1$ and $x_{2}=0$ ( ${\hat{p}}_{10}$ ) in simulated data with sample size n=1000 . The expected proportion of observations with assigned IPC weight of 0 is 5%, 25%, and 50% depending on the censoring distribution. Predictions are derived from a parametric log-logistic survival model (survival), an IPCW-GLM and an IPCW-weighted gradient boosting model (IPCW-gradient-boosting) described as (a) to (c) in Section 3.1. IPC: inverse-probability-of-censoring; IPCW-GLM: inverse-probability-of-censoring-weighted generalized linear model; IPCW: inverse-probability-of-censoring-weighting.

**Figure 4.**
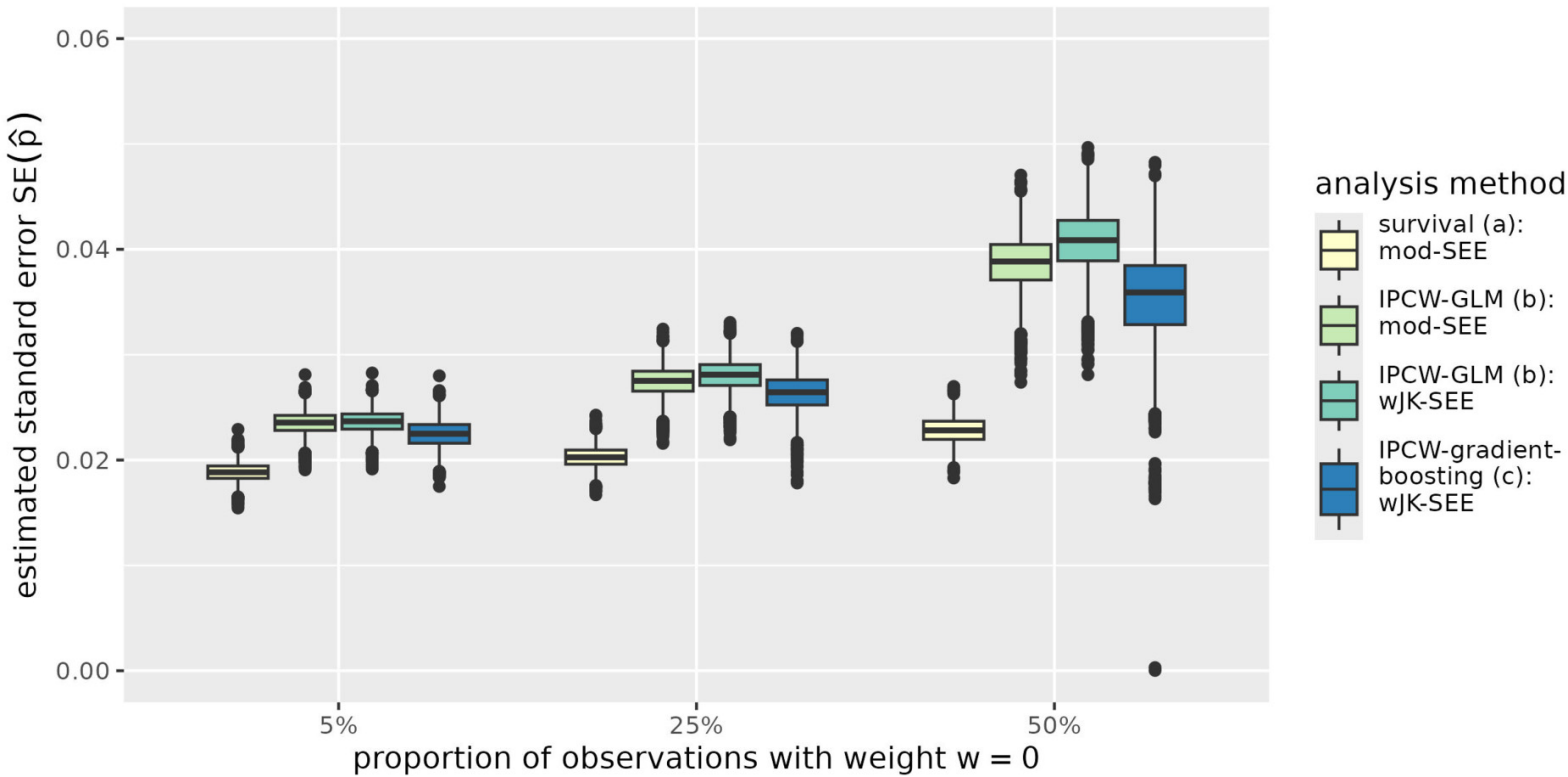
Standard error of the predicted probability to survive time point τ=5 for an individual with $x_{1}=1$ and $x_{2}=0$ ( ${\hat{p}}_{10}$ ) in simulated data with sample size n=1000 . The expected proportion of observations with assigned IPC weight of 0 is 5%, 25%, and 50% depending on the censoring distribution. Standard errors of predictions are derived from the standard errors of regression coefficients using the delta method in the parametric log-logistic survival model and the IPC-weighted GLM (survival:mod-SEE, IPCW-GLM:mod-SEE) and by the adjusted jackknife estimator in the IPC-weighted GLM and the IPC-weighted gradient boosting model (IPCW-GLM:wJK-SEE, IPCW-gradient-bosting:wJK-SEE). The analysis methods (a) to (c) are described in Section 3.1. IPC: inverse-probability-of-censoring; IPCW-GLM: inverse-probability-of-censoring-weighted generalized linear model; IPCW: inverse-probability-of-censoring-weighting.

The results are further illustrated in Figures 1 to 4 exemplarily for the situation of a sample size of n=1000 . The figures illustrate the findings described before: Whereas all methods exhibit unbiased predictions for $p_{11}$ , variability of predictions is higher when IPC-weighted classifiers are applied as compared to the survival model (Figure 1). This can also be observed from Figure 2. Furthermore, Figure 2 also reflects the slightly conservative results of the IPCW-adjusted jackknife estimates of standard error when compared to the model-based estimates of IPC-weighted GLM. For $p_{10}$ , Figure 3 reflects the bias of the IPCW-weighted gradient boosting, that also adversely affects the standard error estimates based on the Jackknife replicates of predictions (Figure 4).

## 4. Application

We illustrate the methods by quantifying the prediction uncertainty of parametric and machine learning classifiers when predicting the individual patient risks of death or graft failure after kidney transplantation. For prediction, modeling data from the German Organ Transplant Registry are used. These data include information on donors, recipients, and the transplantation process of the solid organ transplants performed between 2006 and 2016. Additionally, follow-up information on patient survival and organ failure is incorporated, which hospitals are required to report at least annually. Due to this reporting schedule, it may be more reliable to derive only annual predictions. These predictions will serve to illustrate the methods developed within this article.

Data were filtered to include first-time, kidney-only recipients of deceased donor organs with available information on graft and patient survival data. Donors and recipients above 65 years of age were excluded due to differences in organ allocation for this age group (Eurotransplant Senior Program). This resulted in a sample size of n=10,789 kidney transplantations. We further removed 102 observations with implausible values (weight <30 kg, height <100 cm, years on dialysis >100 years or negative, creatinine >1000 μ mol/L, cold ischemia time > 80 hours or 0 minutes, ABO-incompatible transplants).

The outcome of interest was graft and patient survival probabilities at τ=2,3 , and 4 years after transplantation. Observations with graft failure or patient death within the first 5 days after transplantation ( n=211 ) were excluded, as well as patients with follow-up information <5 days after transplantation ( n=769 ) resulting in a total of 9707 observations. The proportion of censored observations before time τ was 9%,24% , and 54% , respectively.

A Cox proportional hazards model, an IPC-weighted GLM with logit link function and an IPC-weighted gradient boosting classifier were fitted to estimate the graft and patient survival probabilities at time τ . Hyperparameter selection is described in Appendix A. All models were based on the following donor, transplant and recipient factors: donor age, donor height, donor smoking, recipient age, recipient’s years on dialysis, log cold ischemia time, recipient pretransplant blood transfusion, recipient presenting PRA (panel-reactive antibodies), and recipient sex. The details about the variable selection method and handling of missing values are given in Appendix B. For the Cox model and GLM, model-based standard error estimates of predictions were derived, where methods derived by Blanche et al.^12^ are used for the weighted GLM. Additionally, the adjusted jackknife estimator for the standard error of predictions is employed for all models.

Table 8 presents the predictions from the different models exemplarily for a male recipient, of mean age, with mean years of dialysis, without pretransplant blood transfusion, not presenting panel reactive antibodies, receiving a transplant from a non-smoking donor of mean age and height, after the mean log cold ischemia time of the transplant. The predicted patient and graft survival probability at time τ is similar, irrespective of whether a Cox regression approach or an IPC-weighted binary classifier has been applied. The survival probability at 2 years after transplantation is predicted to be ∼ 93% and decreases to around 90% and 87% after 3 and 4 years, respectively. The gradient boosting seems to exhibit a slight bias when the proportion of observations with zero weight is 22.5% or 53.7%. Regarding prediction uncertainty, the application confirms the finding of the simulation studies that IPC-weighted classifiers have higher prediction uncertainty compared to the survival analysis approach that utilizes the full data. The results also indicate that the adjusted jackknife estimate of prediction uncertainty tends to be slightly conservative compared to the model-based approach for GLM. However, when machine learning classifiers such as XGBoost are employed with right-censored data, no model-based approach for prediction uncertainty exists and the proposed adjusted jackknife estimator provides a way to quantify the prediction uncertainty also in these situations.

**Table 8.** Predicted probability with standard error for the graft and patient survival at time τ (years) for the specific patient as described in Section 4.

	% of obs				
τ	with *w* = 0	Method	$\hat{p}$	mod-SE( $\hat{p}$ )	wJK-SE( $\hat{p}$ )
2	8.5	Cox model	0.931	0.0037	–
		IPCW-GLM	0.935	0.0043	0.0044
		IPCW-XGBoost	0.931	–	0.0044
3	22.5	Cox model	0.905	0.0048	–
		IPCW-GLM	0.910	0.0051	0.0058
		IPCW-XGBoost	0.886	–	0.0058
4	53.7	Cox model	0.871	0.0064	–
		IPCW-GLM	0.867	0.0078	0.0080
		IPCW-XGBoost	0.857	–	0.0080

Predictions ( $\hat{p}$ ) are derived from the different prediction models that have been trained on data of the German Organ Transplant Registry. The percentage of observations who were assigned an IPC weight of 0 depends on the censoring rate and thus on τ . For gradient boosting no model-based standard error estimate (mod-SE( $\hat{p}$ )) exists, the survival model does not require IPCW and thus no adjusted jackknife estimator (wJK-SE( $\hat{p}$ )) exists. IPC: inverse-probability-of-censoring; IPCW: inverse-probability-of-censoring-weighting.

**Table 9.** Variables considered in the backward variable selection process to select the variables for prediction modeling of the graft and patients survival probability using data of the German Organ Transplant Registry (Section 4).

Variable	Distinct values
Donor	
Age (years)	–
Creatinine ( μ mol/L)	–
Diabetes	T/F
Height (cm)	–
Hepatitis C antibodies	T/F
Hypertension	T/F
Sex	F/M
Smoker	T/F
Weight (kg)	–
Recipient	
Age (years)	–
Pretransplant bloodtransfusion	T/F
Years on dialysis	–
Height (cm)	–
Hepatitis C antibodies	T/F
Panel reactive antibodies (PRA)	T/F
Sex	F/M
Weight (kg)	–
Transplantation	
Cold ischemia time (minutes)	–
Enbloc transplantation	T/F
Destination	L/A/H/R

Variables considered by Rao et al.^34^ being unavailable or not directly available in the TxReg were:

For the recipient: Race, transplant center, angina pectoris, peripheral vascular disease, drug-treated chronic obstructive, pulmonary disease, and diabetes status.

For the donor: Race, donation after cardiac death, pulsatile perfusion, and year of transplant.

## 5. Discussion

IPCW classifiers have been introduced as a flexible tool for applying in particular machine learning models to survival data. We have proposed a method to consider the IPCW when estimating the uncertainty of individual predictions derived from these classifiers.

Whereas we have focused on classifiers that customize the log-likelihood or loss function using IPCW, bagging with IPC-weighted bootstrapping has been proposed as an alternative.^11^ This approach enables ensemble learning and prediction estimates are expected to be unbiased, following similar arguments as given in Section 2.1. For standard error estimation, the jackknife after bootstrap method^28,29^ could be adapted to incorporate IPC weights, which will be the topic of future work.

Jackknife methods can be computationally challenging in particular for large sample sizes and time-consuming prediction algorithms as for example random forests or deep neural networks. For this reason, our simulation study investigates only a single machine learning algorithm (gradient boosting), which is a limitation. The simulation study is also restricted to a data generation model, which does not reflect non-linear or non-additive effects or a larger number of predictors as for example proposed by Infante et al.^30^ Nevertheless, we chose this simulation model, because for the IPCW-GLM a parametric standard error recently has been proposed^12^ that could serve as a comparator.

Three issues appear with the presented approach similarly to how it does with unadjusted jackknife estimates of standard errors: First, our estimator gives slightly conservative results as typical for the jackknife method.^31^ Second, we rely on the assumption (3) that is similar to the assumption $IF(y_{i})=(n-1)(\hat{p}-{\hat{p}}_{i})$ used in the unadjusted jackknife approach. We demonstrate that assumption (3) holds true in the simplified example given in Section 2.2. However, in more complex scenarios, the influence functions may not be reducible to simpler formulas making this assumption difficult to verify. Third, Jackknife variance estimators are based on predictions from leave-one-out subsamples. Consequently, when the underlying method produces biased predictions—as we observed for the machine learning approach under insufficient sample sizes—this bias can negatively affect the Jackknife variance estimates and, in turn, reduce the coverage probabilities of the confidence intervals.

In situations where IPC weights are close to zero, the proposed adjustment will have less impact on the estimator than in situations with larger weights. This can be observed in the simple tractable example of Section 2.3 where the scaling factor approximates 1 with increasing m and thus with decreasing IPC weights.

Prediction is a common task in statistical and machine learning. We consider reporting the uncertainty of individual risk predictions as crucial, especially when these predictions inform medical decisions. However, there is no firm conclusion on the best measure for reporting individual prediction uncertainties.^32^ The TRIPOD statement^33^ provides guidance on how to report a prediction model, but it does not address how to report the resulting predictions. Recently, Thomassen et al.^17^ proposed the effective sample size as a metric to quantify prediction uncertainty, which may be a more intuitive measure than standard errors or confidence intervals. Although the effective sample size is currently limited to certain parametric prediction models and thus out of the scope of this article, the authors discuss extending it to more flexible models.

It should be mentioned that uncertainty in individual predictions does not only arise from sampling data from the target population. There are other sources of uncertainty, which we have not considered here. One potential source is model misspecification, which is of more concern in statistical modeling than in machine learning due to the flexibility of many machine learning algorithms. Another source is sampling bias. This issue is particularly relevant for machine learning algorithms, which often are trained on large datasets that are more susceptible to sampling bias compared to clinical study data with pre-specified inclusion and exclusion criteria.

## Appendices

### Appendix A. Hyperparameter selection for XGBoost

Hyperparameter optimization for XGBoost was conducted individually for each simulation and for the application to the real data. Candidate configurations were generated using a grid search approach, incorporating both the “gbtree” and “gblinear” boosters. For the “gbtree” booster, different hyperparameter values were explored: for the learning rate, we explored values of $10^{0},10^{-1},\ldots ,10^{-5}$ and for the maximum tree depths we explored values of 12,6,3 , and 1 . The evaluation of candidate configurations was carried out using five-fold cross-validation. To prevent overfitting, early stopping with a patience of 10 iterations was implemented to determine the optimal number of boosting rounds. The performance of each candidate was assessed using the “logloss” metric, which also guided the selection of the best hyperparameter configuration.

### Appendix B. Variable selection

Initial variable selection started with all p= 22 variable candidates as reported by Rao et al.,^34^ being directly available in the TxReg and showing <50% missing values. Missing values in these 22 variables were imputed using the MissForest implementation in missRanger^35^ with 500 trees, a maximum iteration count of 10, sampling of three candidate values and otherwise default parameters. Two nominal variables that exhibited more than 40 different values were excluded. All variables are presented in Table 9. A backward variable selection based on the AIC and employing a Cox proportional hazards model with administrative censoring at year 5 using the fastbw function of the R-package rms (version 6.7.1) selected nine variables, that were finally used in the prediction models (see Section 4).

### Appendix C. Information on the German Organ Transplant Registry data regarding the variables in Table 9

See Table 10.

**Table 10.** Variables considered in the backward variable selection process to select the variables for prediction modeling of the graft and patients survival probability using data of the German Organ Transplant Registry (Section 4).

	Distinct	Variables of TxReg from which information
Variable	values	is extracted
Donor		
Age (years)	–	SPostmBasisAlterDSO, SPostmBasisAlterIQTIG, SPostmBasisGeburtsdatumET
Creatinine ( μ mol/L)	–	SPostmLaborKCKreatininWertET
Diabetes	T/F	SPostmAnamnVorerkrankungenDiabetesMellitus-DSO, SPostmAnamnVorerkrankungenDiabetesMellitusET, SPostmAnamnDiabetesMellitusBeginnJahrET
Height (cm)	–	SPostmBasisGroesseWertDSO, SPostmBasisGroesseWertET, SPostmBasisGroesseWertIQTIG
Hepatitis C antibodies	T/F	SPostmLaborVIRHCVAKRDSO, SPostmLaborVIRHCVAKDSO, SPostmLaborVIRHCVAbET
Hypertension	T/F	SPostmAnamnVorerkrankungenHypertonieDSO, SPostmAnamnVorerkrankungenHypertonieET
Sex	F/M	SPostmBasisGeschlechtDSO, SPostmBasisGeschlechtET
Smoker	T/F	SPostmAnamnRaucherET, SPostmAnamnRaucherPYET, SPostmAnamnRaucherDSO
Weight (kg)	–	SPostmBasisGewichtWertDSO, SPostmBasisGewichtWertET, SPostmBasisGewichtWertIQTIG
Recipient		
Age (years)	–	EBasisGeburtsdatumET
Pretransplantbloodtransfusion	T/F	EBasisBluttransfusionNachRegistrierungET, EBasisBluttransfusionVorRegistrierungET
Years on dialysis	–	WNDialyseBeginnDateET, WNDialyseBeginnDateIQTIG
Height (cm)	–	EBasisGroesseWertET, EBasisGroesseWertIQTIG
Hepatitis C antibodies	T/F	EVirHCVAbET
Panel reactive antibodies (PRA)	T/F	EImmPRAWertET
Sex	F/M	EBasisGeschlechtET, EBasisGeschlechtIQTIG
Weight (kg)	–	EBasisGewichtWertET, EBasisGewichtWertIQTIG
Transplantation		
Cold ischemia time (minutes)	–	TIschaemiezeitKaltWertET, ONQualityFormEvaluierungIschaemiezeitKaltWertDSO
Enbloc transplantation	T/F	ONEnBlocEntnahmeDSO
Destination	L/A/H/R	TBestimmungsortET

Variables considered by Rao et al.^34^ being unavailable or not directly available in the TxReg were:

For the recipient: Race, transplant center, angina pectoris, peripheral vascular disease, and drug-treated chronic obstructive pulmonary disease, diabetes status.

For the donor: Race, donation after cardiac death, pulsatile perfusion, and year of transplant.

### Appendix D. Additional simulation results

Tables 11 to 16 give the simulation results of the simulation study described in Section 3.1 for the predictions for a subject with $\left(x_{1},x_{2})=(-1,1\right)$ and $\left(x_{1},x_{2})=(-1,0\right)$ . For computational reasons results based on $n_{sim}=1000$ simulation runs and include the log-logistic survival model and the IPC-weighted generalized linear model only.

**Table 11.** Mean predicted probabilities to survive time point τ=5 for an individual with $x_{1}=-1$ and $x_{2}=1$ ( ${\bar{p}}_{-11}$ ) in simulated data with sample size *n*.

	% of obs	Parametric	IPCW-generalized
*n*	with *w* = 0	survival model	linear model
200	5	0.596	0.596
200	25	0.596	0.599
200	50	0.595	0.598
500	5	0.596	0.597
500	25	0.594	0.593
500	50	0.594	0.591
1000	5	0.597	0.596
1000	25	0.597	0.597
1000	50	0.597	0.596

The true survival probabaility is $p_{-11}=0.596$ . The expected proportion of observations with assigned IPC weight of 0 is 5%, 25%, and 50% depending on the censoring distribution. IPC: inverse-probability-of-censoring; IPCW: inverse-probability-of-censoring-weighting.

**Table 12.** Standard error of the predicted probability to survive time point τ=5 for an individual with $x_{1}=-1$ and $x_{2}=1$ ( $SE({\hat{p}}_{-11})$ ) in simulated data with sample size n .

		Parametric		IPCW-generalized		
		survival model		linear model		
	% of obs					
*n*	with *w* = 0	SE	mod-SEE	SE	mod-SEE	wJK-SEE
200	5	0.0520	0.0524	0.0605	0.0623	0.0634
200	25	0.0586	0.0574	0.0735	0.0712	0.0740
200	50	0.0692	0.0666	0.1051	0.0957	0.1061
500	5	0.0335	0.0331	0.0390	0.0394	0.0398
500	25	0.0379	0.0364	0.0471	0.0452	0.0464
500	50	0.0422	0.0423	0.0627	0.0615	0.0658
1000	5	0.0237	0.0234	0.0279	0.0279	0.0280
1000	25	0.0256	0.0257	0.0317	0.0319	0.0326
1000	50	0.0297	0.0300	0.0441	0.0433	0.0458

The expected proportion of observations with assigned IPC weight of 0 is 5%, 25%, and 50% depending on the censoring distribution. The empirical standard error over the simulation runs (SE), the mean standard error derived from standard error estimates of regression coefficients using the delta method (mod-SEE) and the mean adjusted jackknife standard error estimates (wJK-SEE) are given for the different prediction models. For gradient boosting no model-based standard error estimate exists, the survival model does not require IPCW and thus no adjusted jackknife estimator exists. IPC: inverse-probability-of-censoring; IPCW: inverse-probability-of-censoring-weighting.

**Table 13.** Coverage probabilities of 95% confidence intervals for the probability to survive time point τ=5 for an individual with $x_{1}=-1$ and $x_{2}=1$ in simulated data with sample size n .

		Parametric	IPCW-generalized	
	% of obs	survival model	linear model	
*n*	with *w* = 0	mod-CP	mod-CP	wJK-CP
200	5	0.963	0.962	0.965
200	25	0.941	0.952	0.962
200	50	0.943	0.942	0.960
500	5	0.952	0.956	0.958
500	25	0.945	0.940	0.947
500	50	0.962	0.953	0.969
1000	5	0.942	0.952	0.953
1000	25	0.954	0.954	0.957
1000	50	0.961	0.947	0.965

The expected proportion of observations with assigned IPC weight of 0 is 5%, 25%, and 50% depending on the censoring distribution. The confidence intervals are derived from confidence intervals on the logit scale, that use the adjusted jackknife standard error (wJK-CP). For comparison, the coverage probabilities when using model-based standard error estimates from the parametric survival and generalized linear model are also given (mod-CP). IPC: inverse-probability-of-censoring; IPCW: inverse-probability-of-censoring-weighting.

**Table 14.** Mean predicted probabilities to survive time point τ=5 for an individual with $x_{1}=-1$ and $x_{2}=0$ ( ${\bar{p}}_{-10}$ ) in simulated data with sample size *n*.

	% of obs	Parametric	IPCW-generalized
*n*	with *w* = 0	survival model	linear model
200	5	0.473	0.472
200	25	0.472	0.473
200	50	0.474	0.468
500	5	0.472	0.472
500	25	0.473	0.472
500	50	0.473	0.469
1000	5	0.471	0.472
1000	25	0.473	0.473
1000	50	0.472	0.470

The true survival probabaility is $p_{-10}=0.473$ . The expected proportion of observations with assigned IPC weight of 0 is 5%, 25%, and 50% depending on the censoring distribution. IPC: inverse-probability-of-censoring; IPCW: inverse-probability-of-censoring-weighting.

**Table 15.** Standard error of the predicted probability to survive time point τ=5 for an individual with $x_{1}=-1$ and $x_{2}=0$ ( $SE({\hat{p}}_{-10})$ ) in simulated data with sample size n .

		Parametric		IPCW-generalized		
		survival model		linear model		
	% of obs					
*n*	with *w* = 0	SE	mod-SEE	SE	mod-SEE	wJK-SEE
200	5	0.0537	0.0536	0.0647	0.0635	0.0647
200	25	0.0583	0.0584	0.0728	0.0735	0.0766
200	50	0.0681	0.0674	0.1011	0.1010	0.1122
500	5	0.0348	0.0341	0.0412	0.0404	0.0408
500	25	0.0390	0.0370	0.0486	0.0465	0.0478
500	50	0.0445	0.0426	0.0676	0.0639	0.0685
1000	5	0.0240	0.0241	0.0277	0.0286	0.0288
1000	25	0.0259	0.0262	0.0326	0.0329	0.0337
1000	50	0.0302	0.0302	0.0448	0.0452	0.0479

The expected proportion of observations with assigned IPC weight of 0 is 5%, 25%, and 50% depending on the censoring distribution. The empirical standard error over the simulation runs (SE), the mean standard error derived from standard error estimates of regression coefficients using the delta method (mod-SEE) and the mean adjusted jackknife standard error estimates (wJK-SEE) are given for the different prediction models. For gradient boosting no model-based standard error estimate exists, the survival model does not require IPCW and thus no adjusted jackknife estimator exists. IPC: inverse-probability-of-censoring; IPCW: inverse-probability-of-censoring-weighting.

**Table 16.** Coverage probabilities of 95% confidence intervals for the probability to survive time point τ=5 for an individual with $x_{1}=-1$ and $x_{2}=0$ in simulated data with sample size n .

		Parametric	IPCW-generalized	
	% of obs	survival model	linear model	
*n*	with *w* = 0	mod-CP	mod-CP	wJK-CP
200	5	0.951	0.952	0.955
200	25	0.952	0.952	0.962
200	50	0.962	0.971	0.981
500	5	0.944	0.933	0.937
500	25	0.942	0.947	0.955
500	50	0.942	0.939	0.954
1000	5	0.951	0.951	0.951
1000	25	0.954	0.961	0.961
1000	50	0.945	0.956	0.964

The expected proportion of observations with assigned IPC weight of 0 is 5%, 25%, and 50% depending on the censoring distribution. The confidence intervals are derived from confidence intervals on the logit scale, that use the adjusted jackknife standard error (wJK-CP). For comparison, the coverage probabilities when using model-based standard error estimates from the parametric survival and generalized linear model are also given (mod-CP). IPC: inverse-probability-of-censoring; IPCW: inverse-probability-of-censoring-weighting.
